# Beta Amyloid Differently Modulate Nicotinic and Muscarinic Receptor Subtypes which Stimulate *in vitro* and *in vivo* the Release of Glycine in the Rat Hippocampus

**DOI:** 10.3389/fphar.2012.00146

**Published:** 2012-07-27

**Authors:** Stefania Zappettini, Massimo Grilli, Guendalina Olivero, Elisa Mura, Stefania Preda, Stefano Govoni, Alessia Salamone, Mario Marchi

**Affiliations:** ^1^Section of Pharmacology and Toxicology, Department of Experimental Medicine, University of GenoaGenoa, Italy; ^2^Department of Drug Sciences, Centre of Excellence in Applied Biology, University of PaviaPavia, Italy; ^3^Centre of Excellence for Biomedical Research, University of GenoaGenoa, Italy

**Keywords:** β amyloid, glycine release, nicotinic receptors, muscarinic receptors, microdialysis

## Abstract

Using both *in vitro* (hippocampal synaptosomes in superfusion) and *in vivo* (microdialysis) approaches we investigated whether and to what extent β amyloid peptide 1–40 (Aβ 1–40) interferes with the cholinergic modulation of the release of glycine (GLY) in the rat hippocampus. The nicotine-evoked overflow of endogenous GLY in hippocampal synaptosomes in superfusion was significantly inhibited by Aβ 1–40 (10 nM) while increasing the concentration to 100 nM the inhibitory effect did not further increase. Both the Choline (Ch; α7 agonist; 1 mM) and the 5-Iodo-A-85380 dihydrochloride (5IA85380, α4β2 agonist; 10 nM)-evoked GLY overflow were inhibited by Aβ 1–40 at 100 nM but not at 10 nM concentrations. The KCl evoked [^3^H]GLY and [^3^H]Acetylcholine (ACh) overflow were strongly inhibited in presence of oxotremorine; however this inhibitory muscarinic effect was not affected by Aβ 1–40. The effects of Aβ 1–40 on the administration of nicotine, veratridine, 5IA85380, and PHA543613 hydrochloride (PHA543613; a selective agonist of α7 subtypes) on hippocampal endogenous GLY release *in vivo* were also studied. Aβ 1–40 significantly reduced (at 10 μM but not at 1 μM) the nicotine-evoked *in vivo* release of GLY. Aβ 1–40 (at 10 μM but not at 1 μM) significantly inhibited the PHA543613 (1 mM)-elicited GLY overflow while was ineffective on the GLY overflow evoked by 5IA85380 (1 mM). Aβ 40–1 (10 μM) did not produce any inhibitory effect on nicotine-evoked GLY overflow both in the *in vitro* and *in vivo* experiments. Our results indicate that (a) the cholinergic modulation of the release of GLY occurs by the activation of both α7 and α4β2 nicotinic ACh receptors (nAChRs) as well as by the activation of inhibitory muscarinic ACh receptors (mAChRs) and (b) Aβ 1–40 can modulate cholinergic evoked GLY release exclusively through the interaction with α7 and the α4β2 nAChR nicotinic receptors but not through mAChR subtypes.

## Introduction

Nicotinic and muscarinic receptors are widely expressed in the brain and implicated in the pathophysiology of many neurological conditions, including Alzheimer’s disease (AD), where typical symptoms include the loss of cognitive function and dementia. The presence of extracellular neuritic plaques composed of β amyloid (Aβ) peptide is a characteristic feature of AD, however, although neurotoxicity is a prominent feature of Aβ, recent data emphasize the existence of synaptic functional roles of this peptide which can modulate the release of several neurotransmitters (see Mura et al., 2012 and references therein). Accordingly, Aβ isoforms and oligomers of increasing molecular dimensions may have different biological actions in a continuum from physiology to pathology, determining loss and gains of function along the course of the disease (Mura et al., 2010a). Indeed, it has been shown that non-neurotoxic Aβ 1–40 concentrations were able to modulate (predominantly, but not exclusively, to inhibit) the release of several neurotransmitters (dopamine, γ aminobutyric acid, aspartate, glutamate) elicited by the stimulation of cholinergic muscarinic and nicotinic receptor [muscarinic ACh receptors (mAChR); nicotinic ACh receptors (nAChR)] subtypes in different brain areas; (Preda et al., 2008; Puzzo et al., 2008; Grilli et al., 2009; Jürgensen and Ferreira, 2010;Mura et al., 2010a,b, 2012; Ondrejcak et al., 2010). The hippocampus, an area which is particularly vulnerable and early target of Alzheimer’s disease and in which the cholinergic pathways are critical for modulation of attention and memory (Parri et al., 2011), Aβ regulates the nicotine-evoked release of both excitatory (glutamate and aspartate) and inhibitory (γ aminobutyric acid) aminoacids (Mura et al., 2012).

Increasing evidence demonstrate that glycine (GLY) is an important aminoacid at hippocampal level which may have a dual role acting as an inhibitory neurotransmitter, when interacting with the strychnine-sensitive receptors, and playing a stimulatory role, when co-activating excitatory *N*-Methyl-d-aspartic acid receptors together with glutamate (Johnson and Ascher, 1987; Luccini et al., 2008; Romei et al., 2009, 2011; Zappettini et al., 2011). No data are available so far on the possible effects of Aβ on the cholinergic receptors which modulate GLY release at the hippocampal level.

In the present study using both *in vitro* (hippocampal synaptosomes in superfusion) and *in vivo* (microdialysis) approaches we investigated whether and to what extent Aβ interferes with the cholinergic modulation of the release of GLY in the rat hippocampus.

The results indicate that (a) the cholinergic modulation of the release of GLY occurs by the activation of both α7 and α4β2 (nAChRs) as previously reported (Zappettini et al., 2011) as well as by the activation of inhibitory mAChRs; (b) the nicotinic modulation of GLY release by α4β2, and α7 nAChRs is inhibited *in vitro* in presence of a nanomolar concentration of Aβ 1–40 which is on the contrary ineffective on the inhibitory mAChR receptor subtypes.

## Materials and Methods

### Animals

Adult male Wistar rats (200–250 g, Harlan, Udine) were used for both *in vivo* experiments and as brain tissue source for *in vitro* experiments. Animals were housed at constant temperature (22 ± 1°C) and relative humidity (50%) under a regular light–dark schedule (light 7 a.m. to 7 p.m.). The *in vitro* experimental procedures were approved by the Ethical Committee of the Pharmacology and Toxicology Section, Department of Experimental Medicine, in accordance with the European legislation (European Communities Council Directive of 24 November 1986, 86/609/EEC) and were approved by Italian legislation on animal experimentation (Decreto Ministeriale number 124/2003-A). The *in vivo* protocol was approved by Ethical Committee of Pavia’s University (registered as 2/2008) according to international regulations for the care and treatment of laboratory animals, to the Italian Act (DL n 116, GU, suppl 40, 18 February, 1992) and to EEC Council Directive (86/609, OJ L 358, 1, 12 December, 1987). All efforts were made to minimize animal suffering and to use the minimal number of animals necessary to produce reliable results.

### *In vitro* experiments

#### Experiments of release

Rats were killed by decapitation and the hippocampus rapidly removed at 0–4°C. Purified synaptosomes were prepared on Percoll^®^ gradients (Sigma-Aldrich, St Louis, MO, USA) essentially according to (Nakamura et al., 1993), with only minor modifications. Briefly, the tissue was homogenized in six volumes of 0.32 M sucrose, buffered at pH 7.4 with Tris–HCl, using a glass-teflon tissue grinder (clearance 0.25 mm, 12 up–down strokes in about 1 min). The homogenate was centrifuged (5 min, 1000 × *g* at 4°C) to remove nuclei and debris; the supernatant was gently stratified on a discontinuous Percoll^®^ gradient (2, 6, 10, and 20% v/v in Tris-buffered sucrose) and centrifuged at 33,500 × *g* for 5 min at 4°C. The layer between 10 and 20% Percoll^®^ (synaptosomal fraction) was collected, washed by centrifugation, and resuspended in physiological HEPES-buffered medium having the following composition (mM): NaCl 128, KCl 2.4, CaCl_2_ 3.2, KH_2_PO_4_ 1.2, MgSO_4_ 1.2, HEPES 25, pH 7.5, glucose 10, pH 7.2–7.4 (Lu et al., 1998). Synaptosomal protein content following purification was 10–15% of that in the supernatant stratified on the Percoll^®^ gradient.

The synaptosomal suspension was layered on microporous filters at the bottom of a set of parallel superfusion chambers maintained at 37°C (Raiteri and Raiteri, 2000; Superfusion System, Ugo Basile, Comerio, Varese, Italy). Synaptosomes were superfused at 1 ml/min with standard physiological medium as previously described. The system was first equilibrated during 36.5 min of superfusion; subsequently, four consecutive 90 s fractions of superfusate were collected and the endogenous GLY content was measured by high performance liquid chromatography as below described. Synaptosomes were exposed to agonists for 90 s starting from the second fraction collected (*t* = 38 min), with antagonists being added 8 min before agonists. The evoked overflow was calculated by subtracting the corresponding basal release from each fraction and was expressed as pmol/mg of synaptosomal proteins. We previously demonstrated that in our superfusion system the indirect drug effects exerted by other mediators in the monolayer of synaptosomes in superfusion are absolutely minimized (Raiteri and Raiteri, 2000).

When studying the release of [^3^H]GLY or [^3^H]Acetylcholine (ACh) hippocampal synaptosomes were incubated for 20 min at 37°C with [^3^H]GLY (final concentration 0.1 μM) in the presence of the selective GLY transporter 1 transporter blocker *N*-[(3R)-3-([1,1′-biphenyl]-4-yloxy)-3-(4-fluorophenyl)propyl]-*N*-methylglycine hydrochloride (final concentration 0.3 μM) or with [^3^H]Choline (Ch, final concentration 0.08 μM). The K^+^-induced overflow from synaptosomes was estimated by subtracting the neurotransmitter content into the first and the third fractions collected (basal release, b1 and b3) from that in the 6-min fraction collected during and after the depolarization pulse (evoked release, b2). The amount of radioactivity released into each superfusate fraction was expressed as a percentage of the total synaptosomal tritium content at the start of the fraction collected (fractional efflux).

### Endogenous GLY determination

Endogenous GLY was measured by high performance liquid chromatography analysis following precolumn derivatization with *o*-phthalaldehyde and resolution through a C18-reverse phase chromatographic column (10 mm × 4.6 mm, 3 μm; Chrompack, Middleburg, The Netherlands) coupled with fluorometric detection (excitation wavelength 350 nm; emission wavelength 450 nm). Homoserine was used as internal standard. Buffers and gradient program were prepared and executed as follows: solvent A, 0.1 M sodium acetate (pH 5.8)/methanol, 80:20; solvent B, 0.1 M sodium acetate (pH 5.8)/methanol, 20:80; solvent C, sodium acetate (pH 6.0)/methanol, 80:20; gradient program, 100% C for 4 min from the initiation of the program; 90% A and 10% B in 1 min; 42% A and 58% B in 14 min; 100% B in 1 min; isocratic flow 2 min; 100% C in 3 min; flow rate 0.9 ml/min.

### *In vivo* experiments

#### Microdialysis probe implantation

Rats were anesthetized with Equithesin 3 ml/kg (pentobarbital 9.7 g, chloral hydrate 42.5 g, MgSO_4_ 21.3 g for 1 l, 10% ethanol, 40% propylene glycol v/v) administered intraperitoneally and placed in a stereotaxic apparatus (David Kopf Instruments, Tujunga, CA, USA). The skin was shaved, disinfected, and cut with a sterile scalpel to expose the skull. A hole was drilled to allow the implantation of the probe into the brain parenchyma. The probe was implanted in the hippocampus (CA1/CA2 regions; AP −5.8 mm, ML ± 5.0 mm from bregma, and DV −8.0 mm from dura) according to the Paxinos and Watson (1986) atlas, and secured to the skull with one stainless steel screw and dental cement. All *in vivo* experiments were performed using microdialysis probes, made in our laboratory according to the original method described by Di Chiara (1990; Emophan Bellco Artificial OR-internal diameter 200 μm, cutoff 40 kDa; Bellco, Mirandola, Modena, Italy), with a nominal active length of 5 mm. Finally, the skin was sutured, and the rats were allowed to recover from anesthesia for at least 24 h before the neurotransmitter release study.

#### Microdialysis samples collection

Microdialysis experiments were performed on conscious freely moving rats. On the day of the experiments (24 h after the surgical procedure), the probe was perfused with artificial CSF containing 145 mM NaCl, 3.0 mM KCl, 1.26 mM CaCl_2_, 1.0 mM MgCl_2_, 1.4 mM Na_2_HPO_4_, buffered at pH 7.2–7.4, and filtered through a Millipore 0.2 μm pore membrane. In all experiments, the microdialysis membrane was allowed to stabilize for 1 h at the flow rate of 4 μl/min, without collecting samples. At the end of the stabilization period, three samples were collected to evaluate baseline release of GLY and then the specific treatment started. All treatments were administered by manually switching syringes and tubing connections to allow drugs diluted in artificial CSF to flow through the probes. Tubing switches were performed taking care to maintain constant flow rates and collection volumes. Both basal and treatment samples were collected every 20 min in 100 μl Eppendorf tubes. The flow rate of 4 μl/min was maintained using a 1000-μl syringe (Hamilton) and a microinjection pump (CMA/100, CMA/Microdialysis AB). *In vitro* recovery of the probe for GLY was about 20%. Each rat was used for only one microdialysis session. At the end of each experiment animals were sacrificed by guillotine, rat brains were removed and the position of the microdialysis probe was verified by histological procedures, slicing the tissues by a cryostat microtome (LEICA CM 1510). Only data from rats in which probe tracks were exactly located in the target area were used for statistical analysis.

#### Immunohistochemical analysis

Immunohistochemical analysis was performed to verify the presence of Aβ in the perfused tissue and to confirm (according to HOECHST 33342 staining) the absence of neurotoxic-induced apoptotic phenomenon. Brain tissue samples were frozen and stored at −80°C. For immunodetection of infused Aβ peptide, 10 μm coronal sections (obtained on a cryostat Leica CM 1510) were incubated with a primary monoclonal antibody recognizing Aβ protein (clone 4G8; Chemicon International). Sections were then incubated with a mouse anti-IgG antibody RPE conjugated (Dako). After the fluorescent labeling procedures, sections were finally counterstained for DNA with HOECHST 33342 and mounted in a drop of Mowiol (Calbiochem, Inalco SpA, Milan, Italy). Fluorescent micrographs were acquired with a Leica TCS SP5 II confocal microscope. After acquisition of fluorescent micrographs, the slides were demounted and then the same sections were slightly counterstained with Mayer hematoxylin, dehydrated, and mounted in DPX for microanatomical analysis. The images were acquired with a BX51 Olympus microscope.

### Statistical analysis

#### In vitro experiments

Multiple comparisons were performed with one-way ANOVA followed by an appropriate *post hoc* test (Dunnett and Bonferroni). Data were considered significant for *p* < 0.05, at least.

#### In vivo experiments

Values were expressed either as amount of GLY measured in the dialyzate (pmol/80 μl) or as area under the curve (AUC), evaluating the cumulative release over time. AUC was used as a measure of treatment exposure and was calculated, for each animal, using GraphPad Prism (version 4.03 GraphPad Software, San Diego, CA, USA), defining as baseline of the area the basal value (average concentration of three consecutive samples immediately preceding the drug dose).

D’Agostino–Pearson Omnibus Test (GraphPad Prism, version 4.03, GraphPad Software, San Diego, CA, USA) and Grubb’s Test (GraphPad QuickCalcs, online calculator for scientists at http://www.graphpad.com/quickcalcs/, GraphPad Software, San Diego, CA, USA) were used as preliminary tests in order to evaluate whether data were sampled from a Gaussian distribution and to detect outliers respectively. All outliers were excluded from the analysis. Data were then analyzed by analysis of variance (one- or two-way ANOVA) followed, when significant, by an appropriate *post hoc* comparison test. Data were considered significant for *p* < 0.05. The reported data are expressed as means ± SEM. The number of animals used for each experiment is reported in the legend to figures.

### Preparation of beta amyloid solutions

In the case of both *in vivo* and *in vitro* experiments, synthetic human Aβ 1–40 (Sigma-Aldrich, Milan, Italy) was dissolved in aCSF at a concentration of 100 mM (stock solution). Then, this solution was filtered through a Millipore 0.2 μm pore membrane and stocked in small aliquots. Working solutions were freshly prepared by diluting an aliquot of Aβ 1–40 stock solution at the final concentrations (10 mM, 1 mM, or 100 nM Aβ 1–40 for *in vivo* experiments, 100 nM, 10 nM, 1 nM, or 100 pM for *in vitro* analysis).

### Chemicals

Beta amyloid (1–40; 40–1), percoll^®^, choline, himbacine, dimethyl sulfoxide, veratridine, nicotine hydrogen tartrate salt (Sigma-Aldrich, St Louis, MO, USA); NFPS (ALX 5407), 5IA85380, PHA543613, AQRA741 (Tocris Bioscience, Bristol, UK); all salts used for the preparation of aCSF (NaCl, KCl, CaCl_2_, MgCl_2_, Na_2_HPO_4_) and for Equithesin (MgSO_4_) were purchased at Merck KGaA, Darmstadt, Germany; chloral hydrate, ethanol 96%, and propylene glycol were used for the preparation of Equithesin and were obtained at VWR BDH Prolabo, Belgium;[^3^H]Choline (specific activity: 60–90 Ci/mmol) and [^3^H]Glycine (specific activity: 15 Ci/mmol) were purchased from Perkin Elmer SpA.

## Results

It is known that Aβ peptides structure and aggregation properties depend on several factors. It is therefore important when using Aβ peptides to specify both the concentration of the soluble Aβ but also the methods used to prepare Aβ solution and to verify the presence of Aβ aggregates (see Parri et al., 2011). This facilitates the interpretation of the results when reporting on Aβ effects and, which is even more important, would facilitate the comparison of data obtained in different laboratories. Hence, in our previous paper we characterized the Aβ peptide conformation we administered *in vivo* by Western Blot procedure (Mura et al., 2012) showing that we administered, at least predominantly, Aβ monomers. We cannot completely exclude by the adopted methods that small amounts of Aβ oligomers are present and may participate to produce the observed effects. In regard to the *in vitro* Aβ preparations, since we did not observe aggregation at the concentrations and the timing (up to 40 min) analyzed *in vivo*, we also do not expect to observe aggregation at the lower concentrations and shorter times used *in vitro* in light of the fact that aggregation is a concentration and time-dependent process.

**Figure 1:**
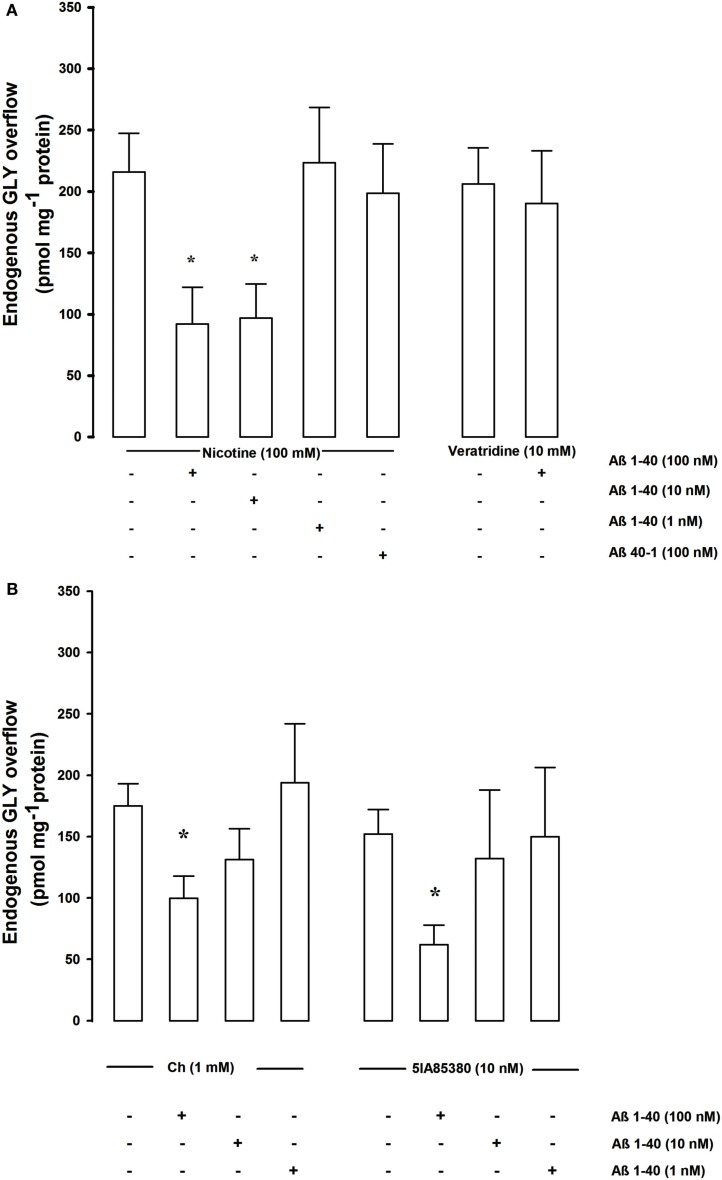
**(A)** Shows the concentration dependence effects of Aβ on nicotine- and veratridine-evoked endogenous GLY overflows from rat hippocampal synaptosomes. Data are mean ± SEM of three to six experiments for each concentration run in triplicate. *p < 0.05 versus nicotine-evoked GLY overflow (one-way ANOVA followed by Bonferroni *post hoc* Test). **(B)** Shows the concentration dependence effects of Aβ on Ch and 5IA85380 evoked endogenous GLY overflows from rat hippocampal synaptosomes. Data are mean ± SEM of three to six experiments for each concentration run in triplicate. *p < 0.05 versus Ch-evoked GLY overflow (one-way ANOVA followed by Dunnett’s Multiple Comparison Test).

Figure 1A shows that the nicotine-evoked overflow of endogenous GLY was significantly inhibited by Aβ 1–40 already at a concentration of 10 nM while increasing the concentration to 100 nM the inhibitory effect did not further increase. The 1-nM Aβ 1–40 concentration was ineffective. The reverse peptide Aβ 40–1 was ineffective even at high (100 nM) concentration. Aβ 1–40 (100 nM) did not affect the veratridine (10 nM)-evoked GLY overflow.

Since it has been shown that two different nAChR subtypes, α7 and α4β2, modulate GLY release (Zappettini et al., 2011), the possibility that Aβ may differentially inhibit the nicotinic control of GLY release has been investigated. In order to verify this point we have studied the effects of two different agonists, Ch and 5-IA85380 hydrochloride (5IA85380), known to act selectively on the α7 and α4β2 nAChR subtypes respectively (Mukhin et al., 2000; Uteshev et al., 2003; Dickinson et al., 2007; Zappettini et al., 2010). Ch (1 mM) and the 5IA85380 (10 nM)-evoked a similar overflow of GLY confirming the involvement of the two receptor subtypes. Both the Ch (1 mM) and the 5IA85380 (10 nM)-evoked GLY overflow were significantly inhibited by Aβ 1–40 at 100 nM but not at 10–1 nM concentrations compared to controls (Figure 1B). Aβ 1–40 (100 nM) did not modify the basal release of endogenous GLY (data not shown).

**Figure 2:**
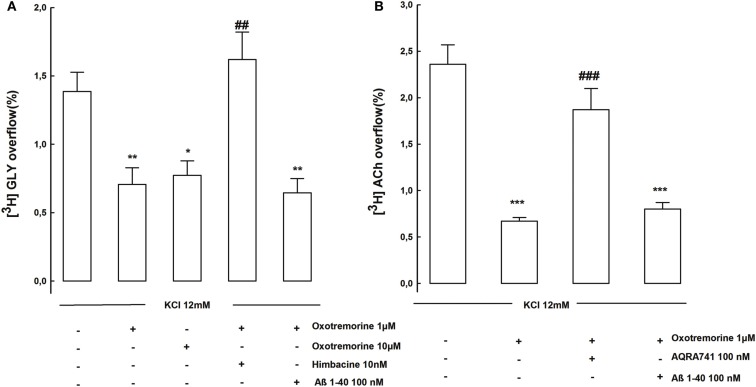
**(A)** Shows the effects of Aβ on muscarinic receptors controlling KCl evoked [^3^H]GLY overflow from rat hippocampal synaptosomes. Data are mean ± SEM of three to six experiments run in triplicate. *p < 0.05; **p < 0.01 versus KCl evoked [^3^H]GLY overflow; ^##^*p* < 0.01 versus KCl plus oxotremorine (1 μM)-evoked [^3^H]GLY overflow. **(B)** Shows the effects of Aβ on muscarinic receptors controlling KCl evoked [^3^H]ACh overflow from rat hippocampal synaptosomes. Data are mean ± SEM of three to six experiments run in triplicate. ***p < 0.001 versus KCl evoked [^3^H]ACh overflow; ^##^*p* < 0.001 versus KCl plus oxotremorine (1 μM) -evoked [^3^H]ACh overflow (one-way ANOVA followed by Bonferroni *post hoc* Test).

It has been demonstrated that also different mAChR subtypes are involved in the modulation of both ACh and GLY release from brain hippocampal synaptosomes (Raiteri et al., 1984; Russo et al., 1993). We investigated whether Aβ was able to affect the muscarinic control of the release of these two transmitters as it was able to disrupt the nicotinic control of GLY release. Figure 2A shows that the KCl evoked [^3^H]GLY overflow was strongly inhibited in presence of oxotremorine. This inhibitory effect was totally antagonized by himbacine but was not affected by Aβ 1–40 (100 nM). In presence of oxotremorine also the KCl evoked release of [^3^H]ACh from hippocampal nerve endings was significantly inhibited (Figure 2B). This result was unexpected but demonstrates quite interestingly that the cholinergic muscarinic modulation of GLY release could be different according to the different brain areas or the different species studied since previous findings using human cortical nerve endings have shown a potentiating effect of oxotremorine on GLY release (Russo et al., 1993). Interestingly also the inhibitory effect of oxotremorine on [^3^H]ACh release was antagonized, as expected, by the specific M2 mAChR antagonist AQRA741 but was not affected by Aβ 1–40 (100 nM; Figure 2B).

**Figure 3:**
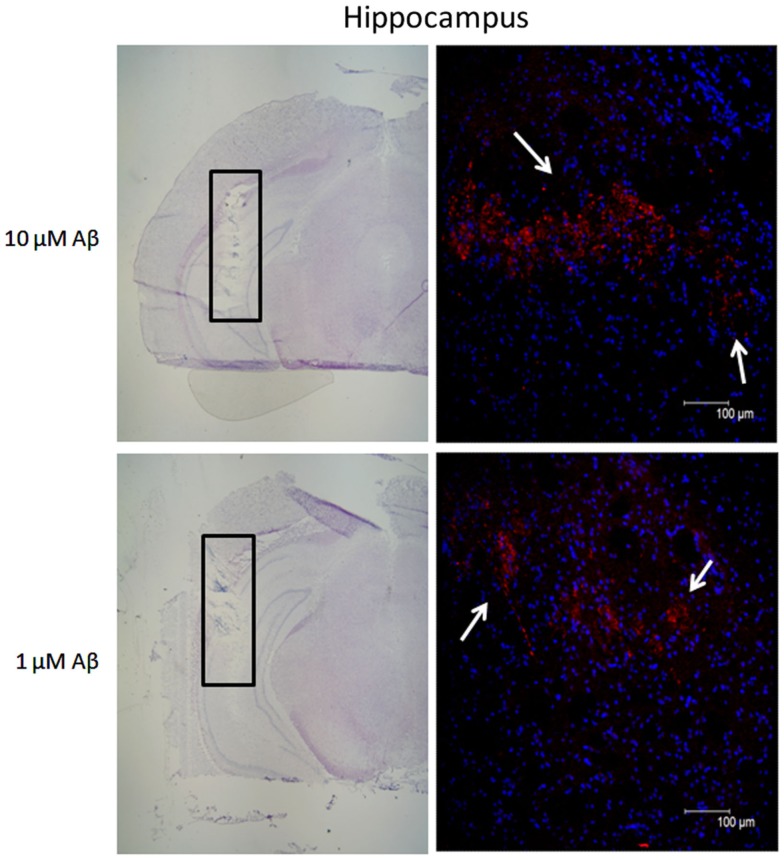
**Immunohistochemical analysis showing the presence of beta-amyloid (Aβ) in hippocampal tissue after the perfusion of the peptide at two different concentrations**. Coronal section indicating the location of the microdialysis probe (hippocampus, black rectangle) counterstained with Mayer Hematoxylin and relative fluorescence micrographs of the area within the black rectangle showing the presence of human Aβ protein. Aβ immunoreactivity (red-PE staining, white arrows) immediately after perfusion of 10 and 1 μM Aβ 1–40. Nuclear DNA was counterstained with Hoechst 33,342 (blue staining). Scale bars for Mayer hematoxylin sections: 200 μM. Scale bars for fluorescent micrographs: 100 μM.

Based on the *in vitro* data we then analyzed the effects of Aβ 1–40 on the administration of nicotine, veratridine, 5IA85380, and PHA543613 hydrochloride (PHA543613, a selective agonist of α7 subtypes) on hippocampal GLY release *in vivo*. In order to test whether the administration of Aβ 1–40 through the dialysis probe allowed the delivery of the peptide to the tissue we performed an immunohistochemical analysis. Figure 3 shows the presence of the peptide for the two concentrations tested *in vivo* (1 and 10 μM) within the hippocampus. Despite the fact that we do not know the exact amounts of Aβ reaching the tissue, there was a visible positive correlation between the concentration administered and the signal of Aβ immunoreactivity in the tissue. Moreover, immunohistochemical analysis shows that no evident signs of apoptosis were observed within the area of amyloid diffusion as shown by Hoechst staining.

**Figure 4:**
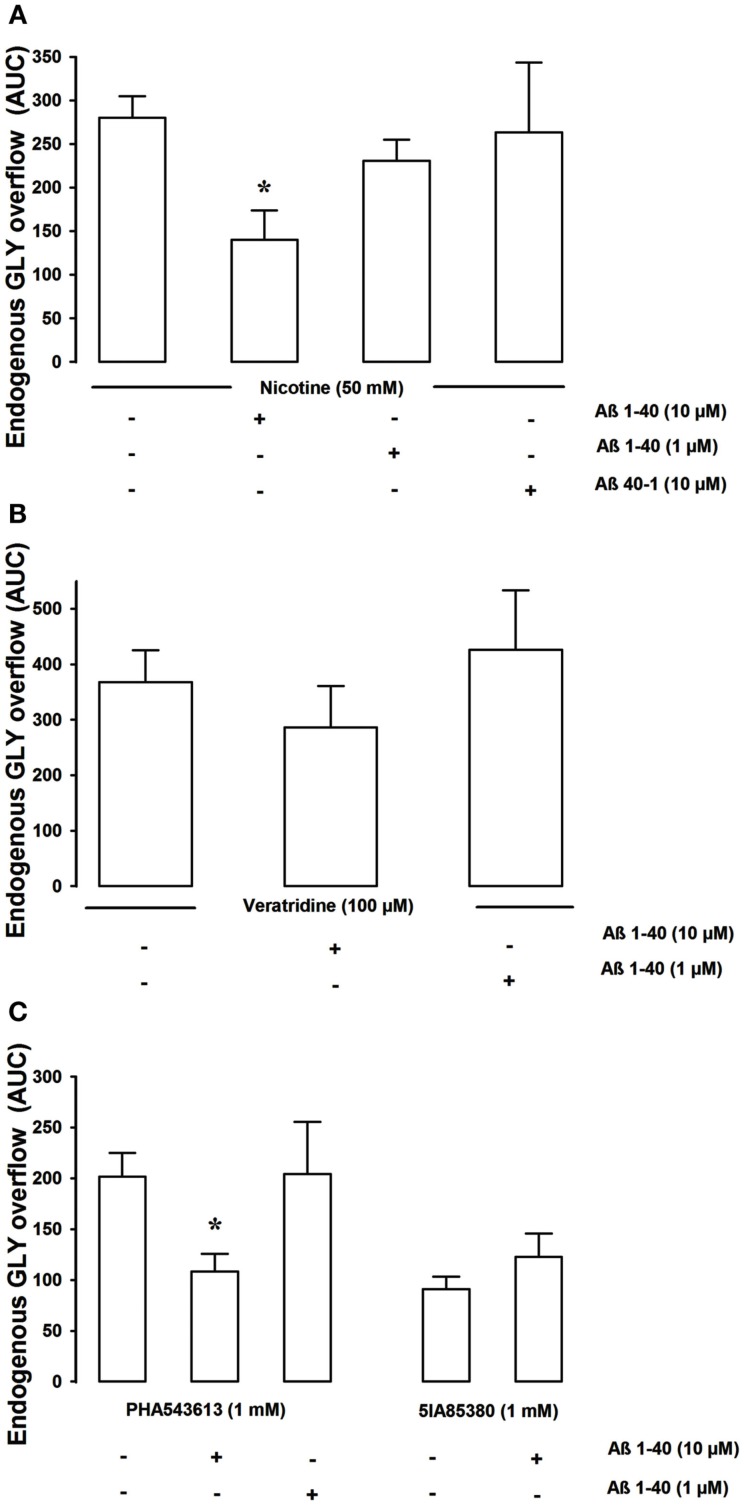
**The figure shows the *in vivo* effect of Aβ 1–40 on the nicotine-, veratrine-, PHA543613-, and 5IA85380-induced overflow of GLY, in rat hippocampus [(A–C) respectively]**. Aβ 1–40 (10 μM but not 1 μM) significantly inhibited the 50-mM nicotine-induced overflow of GLY *p < 0.01 versus nicotine (one-way ANOVA followed by Dunnett’s Multiple Comparison Test). Aβ 40–1 10 μM was ineffective **(A)**. Aβ 1–40 (10 μM) which significantly inhibited the 1-mM PHA543613-induced overflow of GLY was unable to inhibit both the 100-μM veratridine- and the 5IA85380-induced overflow of GLY **(B,C)**. Data are expressed as mean ± SEM of 4–15 individual rats for each experimental group. *p < 0.01 versus PHA543613 (one-way ANOVA followed by Dunnett’s Multiple Comparison Test.

The choice of the concentration of the nicotinic cholinergic agonists to be delivered *in vivo* was derived from previous data demonstrating that the administration by microdialysis of 50 mM nicotine was able to significantly increase the levels of GLY in hippocampal extracellular compartment (Toth, 1996; Fedele et al., 1998; Zappettini et al., 2011). As previously shown in our experimental conditions 40 min-long administration of 50 mM nicotine was able to greatly enhance GLY release from basal values. Figure 4A show that Aβ 1–40 significantly reduced (at 10 μM but not at 1 μM) the nicotine-evoked *in vivo* release of GLY. Aβ 40–1 did not produce any inhibitory effect used at 10 μM concentration. The GLY overflow stimulated by veratridine was unaltered in presence of Aβ 1–40 (Figure 4B). Then we compared the effects Aβ 1–40 after exposure to the selective nAChRs agonists, 5IA85380, and PHA543613. As shown in Figure 4C, Aβ 1–40 (at 10 μM but not at 1 μM) significantly inhibited the PHA543613 (1 mM)-elicited GLY overflow while was ineffective on the GLY overflow evoked by 5IA85380 (1 mM).

## Discussion

It is expected that cognitive deficits and memory impairments in AD patients could be related, at least in part, to Aβ mediated decrease of cholinergic function (Wang et al., 2009a,b, 2010; Parri et al., 2011). However it is still unclear whether these impairments are a consequence of a loss of cholinergic neurons and a decrease of nAChRs or of a direct molecular interaction of Aβ with nAChRs leading to a dysregulation of receptor function or of both mechanisms. To shed some light on these mechanisms we investigated in the present study as well as in previous researches whether Aβ, at concentrations not producing acutely neurotoxicity, is able to disrupt the cholinergic control of neurotransmitter release. To do this we took advantage of the fact that both α4β2, and α7 nAChRs receptors are known to be expressed in rat hippocampal interneurons (McQuiston and Madison, 1999; Sudweeks and Yakel, 2000; Yakel and Shao, 2004), and it is well-known that they have a positive role in regulating cognitive function (Picciotto et al., 1995; Levin and Simon, 1998). Being aware that several neurotransmitters that play important roles in cognitive functions could be affected either directly or indirectly by Aβ, we focused our attention on GLY. Indeed increasing evidence demonstrate that GLY is an important aminoacid at hippocampal level which has a dual role (a) acting as an inhibitory neurotransmitter when interacting with the strychnine-sensitive receptors and (b) playing a fundamental stimulatory role when co-activating excitatory *N*-Methyl-d-aspartic acid receptors together with glutamate (Johnson and Ascher, 1987; Hirai et al., 1996; Luccini et al., 2008; Kubota et al., 2010; Zappettini et al., 2011). The changes in the GLY release may therefore directly interfere with glutamate neurotransmission, which plays an important role in the processes of learning and memory in this brain area.

We here report that the cholinergic modulation of GLY release at the presynaptic level on hippocampal nerve endings was modulated not only by stimulatory α4β2 and α7 nAChRs as previously reported (Zappettini et al., 2011) but also by an inhibitory, mAChR subtype. We do not know whether both nicotinic and muscarinic receptors are present on all nerve endings or they are peculiar of a specific neuronal population and/or their physiological importance in the intact tissue. It is quite interesting however that the two modulatory mechanisms display a different sensitivity to Aβ. The stimulatory effects of both α4β2 and α7 nAChRs were partially blocked by nanomolar concentration of Aβ 1–40 which was on the contrary inactive on the mAChRs inhibiting GLY release. This observation allows to speculate that an excess of Aβ (the range of active concentrations was between 10 and 100 nM *in vitro* and 10 μM *in vivo*) as it may happen because of the disease may dysregulate the cholinergic modulation of hippocampal activity leading to a disproportionate inhibition since it leaves unaffected the muscarinic inhibitory control and impairs the nicotinic stimulatory one. All this occurs in absence of Aβ acutely induced neuronal damage.

The decreased release of GLY may have several functional consequences. Some of them may be relevant to the development of AD pathology. As an example, a decrease of GLY release may reduce the tonic inhibition exerted through the activation of GLY receptors (Mori et al., 2002; Petrini et al., 2004; Farrant and Nusser, 2005) normally providing neuroprotection under pathological conditions, when extracellular GLY levels are elevated (Baker et al., 1991; Saransaari et al., 1997; Zhao et al., 2005; Saransaari and Oja, 2008). A second relevant event caused by a reduced glycine release may consist in a decrease of *N*-Methyl-d-aspartic acid receptors co-activation. At this regard it is important to recall that intracerebroventricular injection of Aβ 1–40 significantly suppress high frequency stimulation-induced LTP (Chen et al., 2006; Wu et al., 2008).

The exact nature of the Aβ interaction with nAChR subtypes is so far not well understood.

Interestingly in our study both the α7 and the α4β2 nAChR subtypes which almost equally contribute to the stimulation of GLY release, were functionally inhibited *in vitro* apparently in a similar extent by Aβ concentrations in the nanomolar range (Figure 2B). However we do not know whether Aβ might have a similar mechanism of action on the two different nAChR subtypes. The inhibitory effect on both the α7 and the α4β2 nAChR was incomplete with a maximal inhibition of about 30–40%. A recently described allosteric binding pocket located within the trans membrane domain of the α7 and of non-α7 nAChRs might provide a potential structure-function mechanism to explain the inhibitory effects of Aβ (Young et al., 2008; Gill et al., 2011, 2012).

Indeed Aβ at concentration in the upper nanomolar range (10 nM) produced full effect while did not produced any effect at 1 nM concentration. Ten nanomolars Aβ beyond the estimated normal concentrations in human CSF but may be caused by the altered precursor protein processing as it occurs along with the disease (Reaume et al., 1996) or be related to defects in the removal of the peptide from the extracellular space as it may occur in the disease in association with the ApoE ε4 genotype (Cramer et al., 2012).

Of course talking about cholinergic modulation of GLY release we have also to consider the possibility that Aβ might for instance interfere directly with the mechanisms which modulate the release of ACh. However our data show that Aβ was ineffective also on the inhibitory muscarinic autoreceptors which inhibit ACh release (Figure 2). We can therefore foresee that in an integrated system, where cellular networks and their functional relationships are completely preserved and several direct and indirect processes are simultaneously taking place in neurons, the effect of Aβ on the cholinergic modulation of GLY function may mostly depend on the interaction of Aβ with the nAChRs.

Our findings *in vivo* largely support this view even if Aβ was unable to inhibit the release of GLY elicited by the specific α4β2 nAChRs agonist while it exerted an inhibition (over 40%) of both nicotine and PHA543613 stimulated GLY release. On the other hand *in vivo* important differences were noticed at the baseline in the ability of the two specific nAChRs agonists to elicit GLY release as compare to *in vitro*. Indeed *in vitro* both α7 and α4β2 nAChRs agonists were able to stimulate to the same extent GLY release; while *in vivo* the release elicited by 5IA85380 was less than a half that obtained in response to PHA543613. *In vivo* to many variables may take place, and in spite of the above described discrepancy, there is a general good agreement of the effects *in vivo* and *in vitro* of Aβ on the release of various neurotransmitters (present work and references) including the observation that so far Aβ has never been able to modify the depolarization (veratridine or potassium)-elicited release of neurotransmitters, but only the one modulated by presynaptic receptors.

In conclusion our findings show that Aβ can modulate cholinergic evoked GLY release exclusively through the interaction with α7 and the α4β2 nAChR nicotinic receptors, acting probably through different mechanisms of action, but not through mAChRs. The present study may therefore provide further insight into the mechanism by which AB impairs synaptic plasticity and cholinergic function in AD brain.

## Conflict of Interest Statement

The authors declare that the research was conducted in the absence of any commercial or financial relationships that could be construed as a potential conflict of interest.
